# The Stromal Vascular Fraction From Fat Tissue in the Treatment of Osteochondral Knee Defect: Case Report

**DOI:** 10.3389/fmed.2018.00154

**Published:** 2018-05-30

**Authors:** Ramil Z. Salikhov, Ruslan F. Masgutov, Mikhail A. Chekunov, Leysan G. Tazetdinova, Galina Masgutova, Oleg V. Teplov, Damir Galimov, Yuri Plakseichuk, Ramil Yagudin, Igor O. Pankov, Albert Rizvanov

**Affiliations:** ^1^Republican Clinical Hospital, Kazan, Russia; ^2^Kazan State Medical Academy, Kazan, Russia; ^3^Institute of Fundamental Medicine and Biology, Kazan Federal University, Kazan, Russia

**Keywords:** mesenchymal stem cells, stromal vascular fraction, osteochondral lesions of the knee, fibrin sealant, fat tissue

## Abstract

In this study we applied autologous fat tissue stromal vascular fraction (SVF) cells in combination with microfracturing technique in a 36-year-old man with an osteochondral lesion of the medial femoral condyle 8 months after the injury. Cell material was generated by fat tissue liposuction from the anterior abdominal wall with subsequent extraction of the SVF and injected through a mini-arthrotomy portal with subsequent fibrin sealant fixation. The follow-up period was 2 years. Clinical score improved from 23 to 96 according to IKDC and from 10 to 90 according to EQ-VAS at 24 months follow-up. Magnetic resonance imaging (MRI) before the surgery revealed an osteochondral lesion with development of significant trabecular edema that remained unchanged for 6 months despite conservative treatment. MRI 1 and 2 years after the surgery showed the recovery of the damaged cartilage thickness with somewhat uneven structure and a decrease in the trabecular edema of the femoral condyle. The use of SVF cells with fibrin sealant fixation might be a promising approach in the treatment of osteochondral joint lesions. Further studies are required.

## Background

Friedenstein and Petrakova were the first to identify and study multipotent stromal cells from fat tissue more than 50 years ago (1). Numerous studies *in vitro* and in animal models showed the possibility of their differentiation in various directions including chondrogenesis. As per an increasing number of publications concerning the clinical use of cell therapy in patients with articular cartilage lesions, especially with knee abnormalities (2–5), such techniques are in high demand. Knee injuries and diseases are among the most frequent reasons for seeking out an orthopedic trauma surgeon or a rheumatologist. There is no exact data on knee articular cartilage lesions, since those are often asymptomatic. These initial llesions can be a trigger for the development of knee osteoarthrosis (6). The use of MRI for the diagnosis of knee injuries showed that meniscal tears and ligament ruptures are frequently combined with articular cartilage lesions. The analysis of collected data from MRI and arthroscopic examinations enabled us to identify a number of traumatic lesion variants including not only the variously deep hyaline cartilage damage, but the lesions of the underlying subchondral bone as well (7). The use of stromal vascular fraction cells is a promising technique for the treatment of hyaline cartilage defects. Our previous studies have shown the effectiveness of SVF cells in the treatment of pseudoarthrosis of the femur (8). In this study we present a case report of successful treatment of a traumatic osteochondral lesion of the medial femoral condyle using autologous SVF cells and fibrin sealant.

### Case presentation

**Figure 1:**
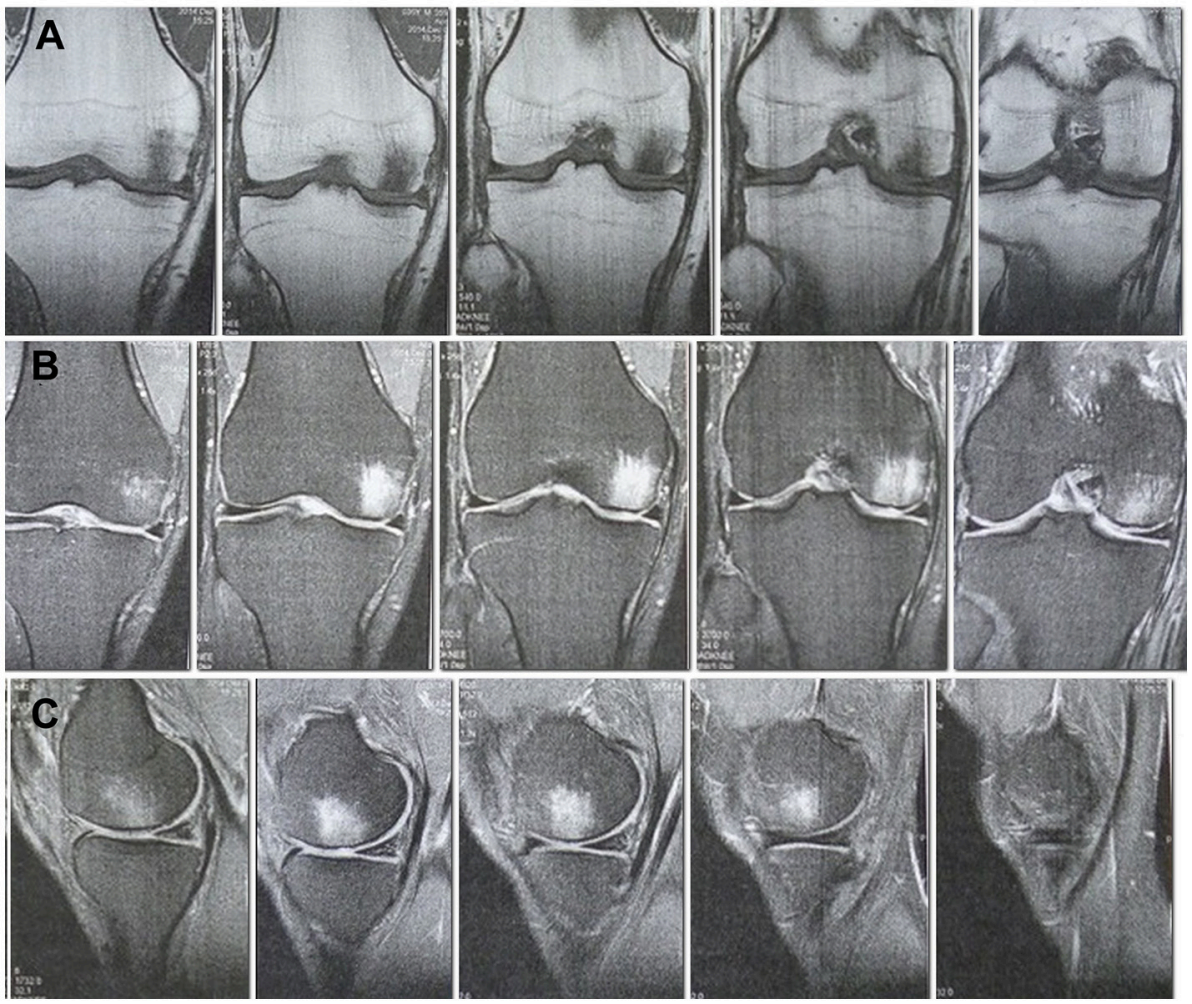
MRI of the right knee 6 months after the injury. A site of local damage to the medial condyle of the thigh is visualized. Pronounced subchondral edema. **(A)** Cor T1FSE. **(B)** Cor PD fat sat FSE. **(C)** Sag PD fat sat FSE.

Patient A., 36, admitted to the orthopedic department No. 1 of the Republican Clinical Hospital (RCH), Kazan, Russia with complaints of pronounced pain in the right knee, getting worse after physical load, for the last 8 months. He sustained an injury on 21.05.14 when skydiving and landed on his straightened lower extremities, a trauma comparable to a fall form a height from ca. 2 m. These were the findings of the MRI (1.5 T) of the right knee carried out on the date of injury: the articular surface of the medial femoral condyle shows a local area of pathological MR signal in the subchondral layer up to a depth of 6 mm with the concomitant marked contusional edema of the medial femoral condyle, the anterior cruciate ligament looks uneven, with inhomogeneous signal due to partial damage, and there is a small amount of fluid in the joint cavity. The MRI of the left knee showed no abnormalities. The patient received a course of conservative treatment (physical therapy, therapeutic exercise, massage) for both knees. The pain subsided in the left knee and persisted in the right. According to the MRI of the right knee (1.5 T) dated 04.12.14, the area of local pathological MR signal in the subchondral part with concomitant marked trabecular edema of the medial femoral condyle remained without significant changes, the excess fluid in the joint was gone (Figures 1A–C). We considered such MRI picture as proof of a traumatic osteochondral lesion of the medial femoral condyle with an underlying significant bone bruise, with high risk of developing osteonecrosis and later progression to posttraumatic osteoarthritis. The clinical examination revealed a tenderness in the medial genicular compartment, the pain intensified during load tests. A significant atrophy of the right thigh muscles was observed. The range of motion in the right knee was 0–100°.

#### Arthtroscopy

On 29.01.15 the arthroscopy of the right knee was performed in the orthopedic department of RCH. A grade 3 local chondromalacia was found in the loaded area of the medial femoral condyle. No lesions of the medial meniscus, lateral meniscus, anterior cruciate ligament, or posterior cruciate ligament were observed.

#### Harvest procedure

The patient was offered a treatment with autologous cells of the stromal vascular fraction as part of an ongoing clinical research project. He gave his written informed consent in accordance with the Declaration of Helsinki. The protocol was approved by the Biomedicine Ethic Expert Committee of Kazan Federal University and RCH (No. 218, 11.15.2012). On 02.02.15 the cell material was collected from fat tissue in the abdominal region by liposuction. Under regional anesthesia, 50 ml of fat tissue were obtained through a 0.5 cm long incision on the anterior abdominal surface using a lipotome. The wound was completely closed with a 5.0 suture, Curafix tight bandage was applied. 1.5 million autologous stromal vascular fraction cells were extracted in a laboratory setting.

#### Stromal vasciular fraction (SVF) extraction

The extraction of autologous SVF cells was carried out by enzymatic disaggregation of fat tissue according to standard protocols published earlier (9). After the incubation, the cells were rinsed 3 times using centrifugation cycles with NaCl at 500 g for 5 min. The sediment was then resuspended with 300 μl of NaCl.

**Figure 2:**
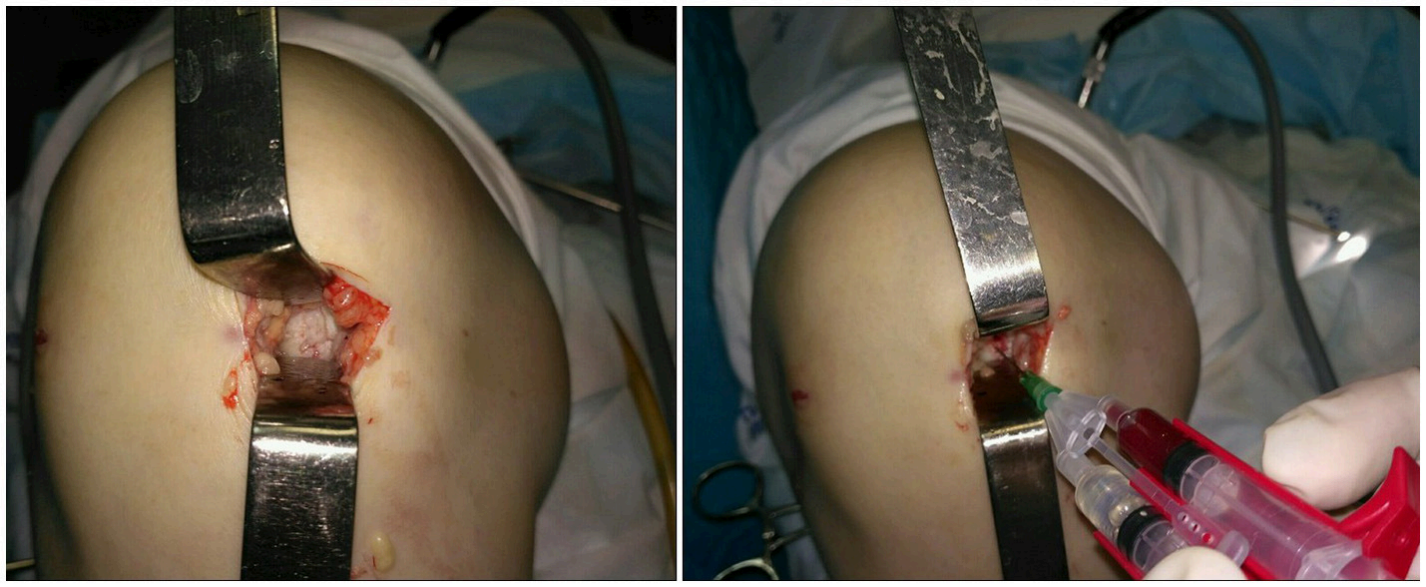
Intraoperative images. Local cartilage damage **(Left)** and stromal vascular fraction transplantation **(Right)**.

On 03.02.15 a 4 cm medial mini-arthrotomy was carried out under spinal anesthesia. The area of the cartilage lesion was ca. 5 cm^2^. Microfracturing of the area was performed. 1.5 million autologous SVF cells were injected together with fibrin sealant Tissucol Kit (fast action) using a duploject system followed by a layered closure of the wound (Figure 2).

### Post-operative care and rehabilitation

The rehabilitation program was divided into 4 periods. In weeks 1–4 walking with crutches with the injured leg bearing not more than half of the body weight and passive motion in the joint up to 90° was allowed. In the weeks 4–8 load up to 80% of the body weight was recommended in combination with wearing of a knee protector, passive extension in the joint and flexion up to 125°, stimulation of thigh and lower leg muscles. In the weeks 8–24 the following activities were allowed: flexion of up to 135°, walking up to 3 km daily, jogging from week 20. From week 24 unlimited motion in the knee joint was recommended, as well as physical activities (swimming, cycling starting from 6 months, aerobics and running from 8 months, team- and contact sports from 12 months after the surgery).

### Analysis methods and outcome measures

To assess the results we used the clinical examination of the patient, the International Knee Documentation Committee (IKDC) score (10) and EuroQol-visual analog scale (EQ-VAS) (11), both max. of 100 points, MRI analysis (1.5 T-pulse sequences: T1 -ax, sag; PD/cor, sag; PD + FSAT cor, sag; T2 + FSAT/cor, sag) before and after the operation, with a follow-up period of up to 24 months.

## Results and discussion

Clinically we observed a gradual recovery of the knee function within 6 months. Acute pain subsided. There were no signs of instability. At 6 months follow-up the range of motion in the right knee joint was 0–140° with no signs of instability. The patient was satisfied with the treatment outcome despite periodically occurring pain during sports activities, which did not prevent the patient return to the previous level of physical activity.

**Figure 3:**
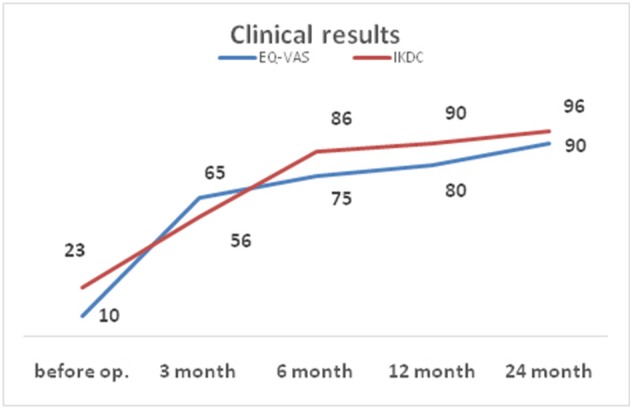
Clinical outcomes according to IKDC and EQ-VAS scores during 24 months after treatment.

The IKDC score before operation was 23, at 3 months follow-up it was 56, at 6 months 86, at 12 months 90, at 24 months follow-up −96. On examination 2 years after the surgery the patient reported the occurrence of pain during some sporting activities, such as skiing or playing soccer, while performing jumps with the operated extremity. No knee edema was observed. The preoperative EQ-VAS score was 10, progressing to 65 at 3 months follow-up, 75 at 6 months, 80 at 12 months and 90 at 24 months follow-up (Figure 3).

**Figure 4:**
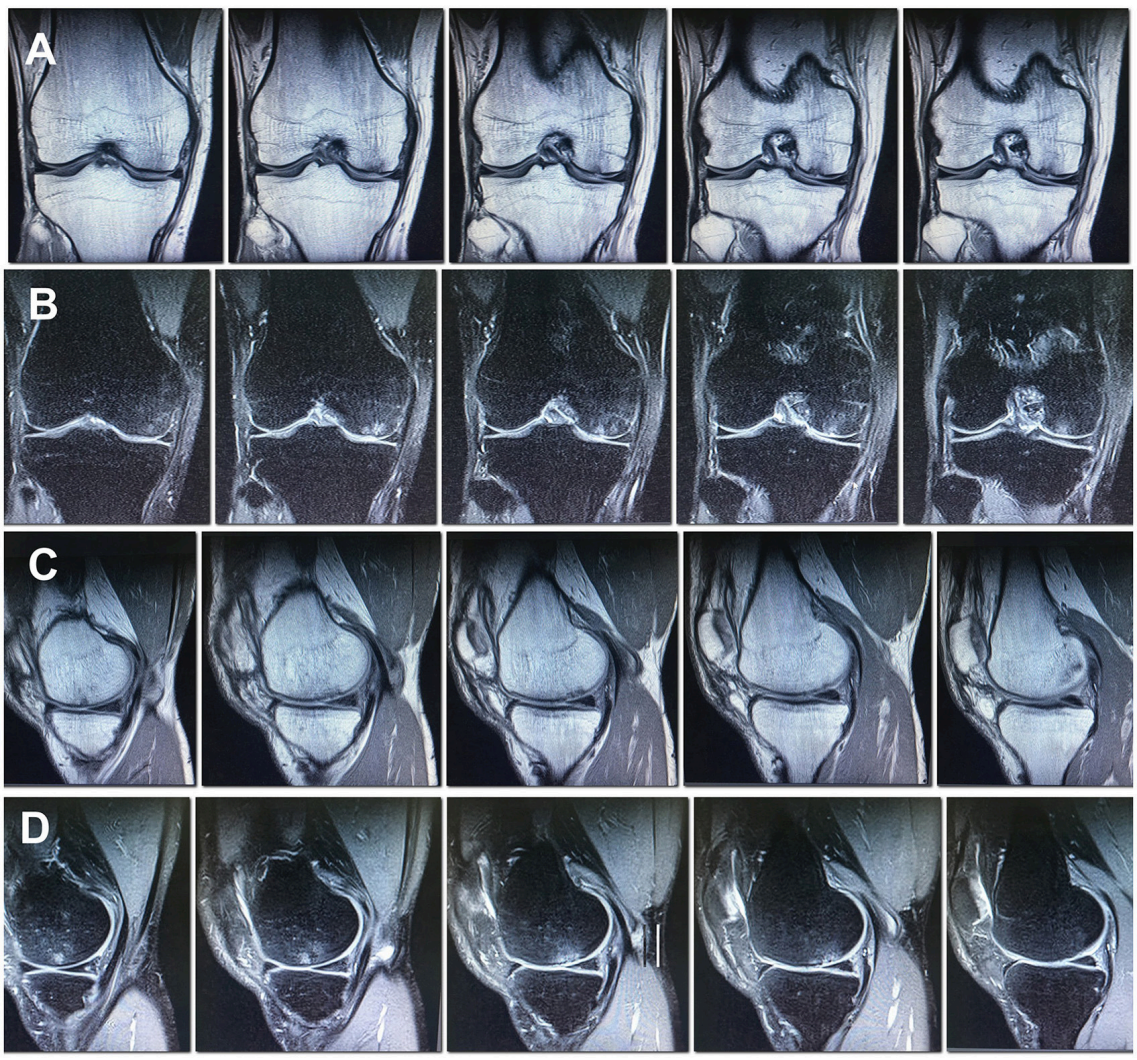
MRI of the right knee 10 months after the surgery. Decreased subchondral edema. **(A)** Cor T1FSE; **(B)** Cor PD fat sat FSE; **(C)** Sag T1FSE; **(D)** Sag PD fat sat FSE.

According to MRI findings 10 months after the surgery the area of local pathological MR signal in the subchondral compartment decreased substantially, almost complete resolution of the trabecular edema was observed, the hyaline cartilage in the area of the defect was thinned and showed inhomogeneous structure (Figures 4A–D).

**Figure 5:**
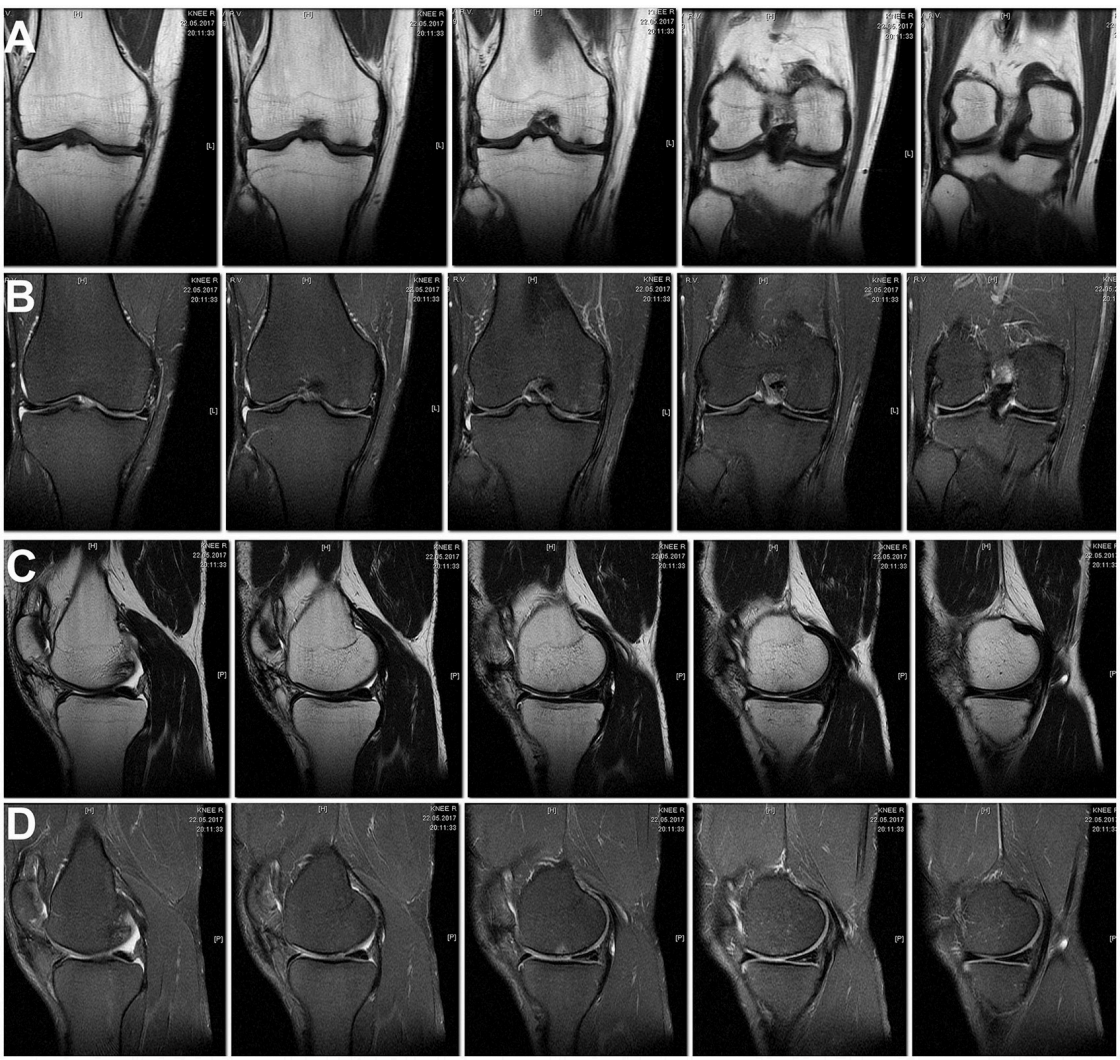
MRI of the right knee 27 months after the surgery. The hyaline cartilage slightly inhomogeneous structure, with restored thickness, in the subchondral compartment an area of osteosclerosis in the form of a small local pathological MR signal. **(A)** Cor T1FSE; **(B)** Cor PD fat sat FSE; **(C)** Sag T1FSE; **(D)** Sag PD fat sat FSE.

In the MRI 27 months after the surgery, the structure of the hyaline cartilage was still inhomogeneous, but with restored thickness, and an area of osteosclerosis in the form of a small local pathological MR signal has formed subchondral (Figures 5A–D).

As acknowledged by many authors, the treatment of hyaline cartilage lesions is a difficult task that requires a complex approach involving a number of biological and biomechanical techniques. Today it is apparent that such technically simple surgical procedures as abrasion chondroplasty, microfracture, or osteoperforation are suitable mainly for small defects. A number of factors may affect clinical outcome including age, preoperative intervals, body mass index, higher preoperative activity levels, and lesion size (<4 cm^2^) (12).

Research into the transplantation of autologous cultured chondrocytes (ACI technique), known since 1980, demonstrated good experimental and clinical outcomes for large-sized defects; moreover, the restoration of hyaline cartilage was noted (13). Today this procedure cannot be considered widely available due to its high price and long-term multiple-stage treatment. Besides, during chondrocyte cultivation multiple cell passages are performed with consequent hazard of possible cell transformation and contamination.

The Autologous Matrix-Induced Chondrogenesis (AMIC) technology based on supporting migration of autologous mesenchymal cells by means of microfracture of the subchondral bone in the area of cartilage defect contained by a collagen membrane is a remarkable example of combined biotechnological and biological approaches (14). Due to its relative simplicity and good clinical outcomes this procedure took the lead in the treatment of local defects of the articular cartilage larger than 1 cm^2^ in size. However, the use of cell-free scaffold in case of osteochondral defects does not lead to the complete solution of the problem, since in spite of substantial improvement in the state of such patients at medium-term follow-up, changes in the subchondral layer and in the bone as a whole cannot be considered satisfactory (15). Most likely there are biological hindrances to the migration of the sufficient quantity of autologous mesenchymal cells through the site of damaged, partially avital subchondral bone, especially when the local situation is worsened by trabecular edema. In such case it is necessary to create conditions for the delivery of multipotent cells for the recovery of cartilage and bone tissue to the injured area. One of such solutions is the use of autologous multipotent mesenchymal cells from fat tissue.

The advantages of using fat tissue-derived MSCs include the ability to harvest sufficiently large amount of cell material, its availability and biological safety. Although adipose-derived MSCs demonstrated a smaller response to chondrogenetic induction *in vitro* as compared to cells derived from the bone marrow (16), their effective differentiation after addition of a number of factors such as bone morphogenetic protein BMP-6, dexamethasone, TGF-β1, and IGF-1 was observed (17). The lipoaspirate from the fat tissue that is used in clinical practice is very similar to so called stromal vascular fraction (SVF) which beside the MSCs contains a number of other cell types that also stimulate regeneration and revascularization, demonstrate anti-inflammatory and immunomodulatory properties (18). MSCs as part of the SVF from the fat tissue or bone marrow are currently successfully used for treatment of cartilage defects and osteoarthrosis of knees and other joints by injecting or applying them with carriers (19–21).However, there are only few studies devoted to the use of MSCs in case of osteochondral lesions of the knee.

For example, Adachi et al. (22) reported a successful treatment of a 21-year old man with a large osteochondral defect in the knee joint after septic arthritis using mesenchymal stem cells (MSCs) from the bone marrow on a porous hydroxyapatite ceramic (IP-CHA) carrier (22).

The study of Buda et al. (23) included the implantation of bone marrow-derived cells cultured on a scaffold (Hyalofast) with platelet-rich fibrin. The treatment outcomes of 20 patients with osteochondral defects of the medial or lateral femoral condyles showed a correlation between good clinical outcomes and MRI findings confirmed by high levels of type II collagen on histological examination (23).

In the reported case we used mini-arthrotomy for the precise delivery of the cell material and its fixation with the help of the fibrin sealant. Thus, we suggest that SVF cells attached by fibrin sealant at the site of a cartilage lesion played one of the major roles in its recovery, which distinguishes our method from others, where the cells are simply injected into the joint cavity. Although the quantity of derived cells (1.5 million) in case of our patient was relatively small because of limited fat tissue availability, it was enough for the stimulation of regenerative processes.

In the presented case report, the patient had a pronounced trabecular edema of the medial condyle of the femur, which didn't decrease for a period of 6 months since the injury, a sign of poor prognosis. We believe that the microfracturing performed decreased the trabecular edema and contributed to the migration of implanted stem cells to the area of the osteochondral lesion. At the same time, the use of only microfracturing with a large amount of osteochondral damage (5 cm^2^) could hardly provide a positive result (restoration of the cartilaginous tissue) within a period of 24 months. A recently published study by Nguyen et al. (24) showed that arthroscopic microfracturing with SVF injection significantly improved WOMAC, Lysholm, and VAS scores over the entire 18-month study period. MRI findings showed that the regenerated cartilage layer of patients treated with arthroscopic microfracturing and SVF was thicker at 12 and 18 months after the procedure. At a period of 18 months, these results were significantly better than in a cohort with arthroscopic microfracturing without SVF (24).

In our study, MRI 1 and 2 years after the surgery showed thickness recovery of the damaged cartilage, decrease in the trabecular edema of the femoral condyle, ant the appearance of osteosclerosis in the subchondral compartment of the injury site in the form of a small focus of local pathological MR signal. These data provide evidence of still ongoing reparative processes in the cartilage and bone tissue even 2 years after the surgery. To date, there is no unified MRI protocol, which allows estimate the degree of restoration of damaged hyaline cartilage. First of all, this is due to the pronounced differences in the parameters of the MRI devices used and their rapid technological improvement. Isotropic 3-D sequences show great promise for improving cartilage imaging and also for the diagnosis of surrounding pathologies within the knee joint (25). Quantitative/biochemical MR approaches are able to provide a specific measure of the composition of cartilage (25). Morphological MRI provides the basis for diagnosis and follow-up evaluation of cartilage defects and surgical cartilage repair, whereas biochemical MRI provides deeper insight into the composition of cartilage and cartilage repair tissue (25). In the same time standard morphological approaches can demonstrate the constitution of cartilage repair tissue (25).

Following autologus chondrocyte implantation (ACI), MRI can help define the defect fill, the integration of the graft with the underlying bone and adjacent native cartilage, and the status of the subchondral bone plate and bone marrow (26). In cases of incomplete defect fill, MR can demonstrate underfilling of the repair site either in focal areas or as an overall thin cartilage. In Buda et al. (23) study of treatment 20 patients with osteochondral defects by bone marrow MSCs imaging sequences were carried out according to the cartilage repair tissue grading scale (the MOCART scoring system), which includes 9 parameters, such as degree of defect repair, integration to the surrounding cartilage, surface of the repaired tissue, structure of the repaired tissue and subchondral bone adhesions effusion (23). Complete degree of defect repair was observed in 14 patients, in 4 were hypertrophies, 2 incomplete coverage. Complete integration with the surrounding cartilage was in 16 cases, in 4 incomplete. The cartilage structure was homogeneous in 6 patients, in 14 inhomogeneous. Subchondral plate and subchondral bone were changed in 14 patients. We suggest, that estimation of the thickness and structure of cartilage in the damage area, its integration with intact cartilage, assessment of the condition of the subchondral bone are important parameters that allow us to evaluate the degree of recovery of osteochondral injury.

## Conclusions

The clinical outcomes and MRI data of the patient with a local traumatic osteochondral lesion of the medial femoral condyle with the follow-up period of more than 2 years demonstrate the possibility of successful use of SVF from fat tissue for treatment of full-thickness cartilage defects associated with subchondral bone lesion and significant trabecular edema. There are a number of limitations with this study including that the introduction of SVF with fibrin sealant was used in combination with the microfracturing procedure. However, isolated microfracturing leads to the formation of fibrous cartilage, which is mechanically inferior to hyaline. Our technique is an attempt to obtain hyaline-like cartilage as in the ACI procedure, using less funds and shortening the treatment time. In this case report, the use of SVF from fat tissue allows a large number of MSC sells to be delivered to the site of cartilage defect, and the fibrin glue serves as a matrix. Revision arthroscopy and histological examination could provide more information to confirm our results. Further clinical trials are needed with larger number of case studies using autologous SVF in patients with local osteochondral lesions of the knee and other joints, probably using additional techniques facilitating further proper restoration of the cartilage and bone tissue.

## Consent for publication

Written informed consent was obtained from the patient for publication of this case report and accompanying images.

## Author contributions

RS performed the experimental the work and data analysis, contributed to data interpretation and wrote the manuscript. RM was responsible for the overall study concept and the final manuscript. RS, RM, DG, and OT performed the experiments. LT and GM isolated stromal vascular fraction from fat tissue. YP, RY, IP, and AR scientific discussion. All the authors contributed to manuscript writing and approved the submitted manuscript.

### Conflict of interest statement

The authors declare that the research was conducted in the absence of any commercial or financial relationships that could be construed as a potential conflict of interest. The reviewer TN and the handling Editor declared their shared affiliation.
